# Structure and mechanism of membrane transporters

**DOI:** 10.1038/s41598-022-17524-1

**Published:** 2022-08-02

**Authors:** Lan Guan

**Affiliations:** Department of Cell Physiology and Molecular Biophysics, Center for Membrane Protein Research, School of Medicine, Texas Tech University Health Sciences Center, Lubbock, TX USA

**Keywords:** Biochemistry, Structural biology

## Abstract

Membrane transporters are an important group of proteins in physiology and disease. Their functions make them common drug targets, but their location in the lipid bilayers poses a tremendous challenge to researchers. The current stage of development of structural biology, in addition to new research tools, has largely facilitated the acquisition of knowledge about transporters and mechanisms. This Collection presents recent studies, covering bioenergetics, structure and functional characterization of various transporters, lipids-protein interactions, and novel research tool development.

Membrane-embedded transporters play essential roles in mediating the uptake of vital nutrients, and the removal of unwanted substances across the cell membranes, to maintain their critical concentrations. Their function and dysfunction are also associated with various diseases, such as obesity and cancers. Transport bioenergetics and mechanisms are therefore crucial for developing novel therapeutic drugs for treatments, and transporters can be used for effective drug delivery into cells or across critical barriers such as the blood/brain barrier. However, the hydrophobic nature of transporters poses significant problems for research, so the knowledge of membrane proteins generally lags far behind that of soluble proteins. In recent years, the availability of high-resolution 3-D structures determined by cryoEM techniques and crystallography has greatly facilitated the studies of transporters and their mechanisms of action. This Collection is dedicated to this line of research.

Peter Mitchell’s chemiosmotic hypothesis, proposed in 1961, transformed the field of bioenergetics and transport^1^. The hypothesis stated that the electrochemical H^+^ gradient, the proton motive force (PMF), is the direct energy source for ion-coupled secondary active transport. This has been systematically and experimentally verified by H. Ronald Kaback^2^ and many other researchers.

As this implies, active transport can be categorized into primary and secondary active transport. The primary active transporters utilize the chemical energy from ATP hydrolysis. The secondary active transporters use the pre-established H^+^ or Na^+^ electrochemical ion gradients to move substrates against concentration gradients. Recently, these transmembrane-electrostatically localized protons (TELP) have been recognized as a primary contributor to Mitchell’s PMF^3^. In addition, a follow-up study in this Collection identified a significant thermotropic component of PMF^4^. Lee found that mitochondria can isothermally utilize environmental heat through TELP to drive the synthesis of ATP, thus locking substantial amounts of the heat energy into ATP molecules. This work has refined and improved our knowledge of transport bioenergetics.

Mitchell also conceptualized a “mobile barrier” hypothesis to explain how the transporters mediate substrate translocations across proteins, and how the binding of substrate(s) dictates conformational changes^5^. Over the past decades, this hypothesis, also described as alternating-access actions, has been largely tested and confirmed by a battery of studies in the field, including in high-resolution structures, molecular dynamics (MD) simulations, and biophysical analysis^6–9^. Furthermore, cooperative binding between two transported solutes has also been recognized as the core mechanism for secondary active transport^10,11^.

In this Collection, MD simulations provide a new understanding of the kinetic coupling of ions and the conformational changes of Mhp1, a member of the nucleobase:cation symporter-1 family^12^. The DEER distance measurements of a major facilitated superfamily exporter MdfA^13^ also offered an original insight into the conformational dynamics of this antiporter. Mutations at the ion-binding sites have often been found to cause fascinating effects, such as an asymmetric effect on Na^+^ and K^+^ binding to NaK-ATPase^14^, and altered stoichiometry in a NhaB Na^+^/H^+^ exchanger^15^. Specific elimination of Na^+^ binding from a common binding for H^+^, Na^+^, and Li^+^ was also reported in a melibiose transporter MelB^16^.

Beyond the interaction between transporter and substrate, the importance of the interactions between the transporter and the surrounding lipid bilayer has also been well recognized^17^ and is a recent hot research topic. Two articles stressed the important roles of cardiolipin in the stability and functions of a bacterial translocon^18^ and a NhaA Na^+^/H^+^ antiporter^19^, respectively. Another study characterized how the interaction between an Arg residue and phospholipids modulates the properties of a sarcoplasmic reticulum Ca^2^^+^-ATPase^20^.

This Collection also recruited several studies on research tools. Membrane protein research often faces problems in identifying proper detergents due to difficulties with solubility. The high-throughput detergent studies in this Collection provide a large body of data on detergent effects on the stability of membrane proteins by determining two related parameters of protein unfolding and aggregation. It is noted that some detergents, such as phosphocholine detergents, could afford opposite effects on the two parameters^21^. Recently, the lipid nanodiscs, which retain a lipid-bilayer core around membrane proteins and provide a more native-like environment than detergent micelles, have been shown to be a useful tool^21^. One article described a new class of copolymers that can extract membrane proteins directly as a form of lipid nanodiscs without the required membrane scaffold proteins^23^. Another study described a cell-free expression tool to study the co-translational folding of transporters without the presence of translocon insertase^24^.

Emerging techniques, including the cryoEM method and the artificial intelligence-based AlphaFold 2, have rapidly advanced our understanding of membrane transporters in a short period, and we expect continued growth of our knowledge. While this editorial cannot hope to discuss these important developments in detail, I hope it draws your attention to the Collection, and the recent progress in membrane transporter research.
